# Correction to: Pharmacokinetic and Pharmacodynamic Studies of Elacestrant, A Novel Oral Selective Estrogen Receptor Degrader, in Healthy Post-Menopausal Women

**DOI:** 10.1007/s13318-020-00638-0

**Published:** 2020-08-30

**Authors:** Maureen G. Conlan, Erik F. J. de Vries, AWJM Glaudemans, Yamei Wang, Steven Troy

**Affiliations:** 1 Department of Oncology Clinical Development, Radius Health, Inc., 950 Winter Street, Waltham, MA 02451 USA; 2 Department of Nuclear Medicine and Molecular Imaging, University Medical Center Groningen, University of Groningen, Hanzeplein 1, 9713 GZ Groningen, The Netherlands; 3 Department of Biostatistics, Radius Health, Inc., 950 Winter Street, Waltham, MA USA; 4 Department of Clinical Pharmacology, Radius Health, Inc., 950 Winter Street, Waltham, MA USA

## Correction to: European Journal of Drug Metabolism and Pharmacokinetics 10.1007/s13318-020-00635-3

Authors would like to correct the errors in table 2. Corrected version of Table 2 given below.

**Table 2:** Pharmacokinetic parameters of elacestrant in Study 001 and Study 004

Study 001							
SAD part^a^	*C*_max_ (ng/ml)	*t*_max_ (h)	AUC_0–last_ (ng·h/ml)	AUC_0–∞_ (ng·h/ml)	*t*_1/2_ (h)	CL/F (l/h)	*V*_*z*_/*F* (l)
1 mg IV (*n* = 5)	69.8 ± 30.7	0.04 ± 0.02	23.9 ± 3.6	28.0 ± 5.2	33.4 ± 6.2	36.8 ± 7.7^d^	1730 ± 155^e^
1 mg (*n* = 2)^b^	0.06 ± 0.02	2.63 ± 2.65	NR	NR	NR	NR	NR
10 mg (*n* = 6)	0.7 ± 0.4	1.92 ± 1.28	13.1 ± 9.4	16.8 ± 11.7	31.9 ± 8.1	760 ± 323	33,700 ± 13,800
25 mg (*n* = 6)	1.8 ± 0.5	1.64 ± 1.42	32.2 ± 15.7	40.6 ± 21.6	32.5 ± 6.0	786 ± 419	34,500 ± 13,300
50 mg (*n* = 6)	3.4 ± 0.7	1.92 ± 2.03	61.3 ± 11.7	73.3 ± 13.2	29.1 ± 3.4	702 ± 134	29,800 ± 8910
50 mg fed (*n* = 6)	7.0 ± 1.5	4.17 ± 1.33	96.8 ± 20.0	116 ± 26.9	28.8 ± 2.7	451 ± 111	18,500 ± 3050
100 mg (*n* = 6)	11.8 ± 2.0	2.58 ± 1.72	247 ± 71.9	294 ± 80.8	28.4 ± 3.9	361 ± 92	14,900 ± 4920
200 mg (*n* = 6)	31.5 ± 5.6	3.25 ± 1.57	649 ± 183	774 ± 239	27.4 ± 3.7	281 ± 90	10,800 ± 2860

MAD part^a^	*C*_max_ (ng/ml)	*t*_max_ (h)	AUC_0–τ_ (ng·h/ml)	*R*_ac_	*t*_1/2_ (h)	CL_ss_/*F* (l/h)	*V*_*z*_/*F* (l)
10 mg/day (*n* = 7)—day 1	0.5 ± 0.1	2.25 ± 2.24	5.6 ± 2.0				
Day 7	0.8 ± 0.2	0.97 ± 0.27	10.8 ± 3.7	1.95 ± 0.29	37.9 ± 4.7	1020 ± 347	54,600 ± 15,100
25 mg/day (*n* = 7)^c^—day 1	1.5 ± 0.5	2.92 ± 3.50	16.0 ± 4.1				
Day 7	2.6 ± 0.8	1.29 ± 0.47	35.3 ± 13.9	2.20 ± 0.68	41.1 ± 12.7	799 ± 281	47,100 ± 21,100
50 mg/day (*n* = 8)—day 1	4.5 ± 1.0	1.72 ± 0.84	45.2 ± 12.8				
Day 7	5.7 ± 1.2	2.78 ± 2.47	82.1 ± 17.3	1.86 ± 0.27	31.1 ± 6.8	634 ± 139	28,300 ± 7750
100 mg/day (*n* = 8)—day 1	10.4 ± 2.8	3.93 ± 3.01	122 ± 55.6				
Day 7	20.5 ± 7.7	2.50 ± 1.10	265 ± 118	2.18 ± 0.37	35.5 ± 8.2	437 ± 166	22,500 ± 10,100
200 mg/day (*n* = 8)—day 1	27.3 ± 6.8	2.94 ± 1.47	284 ± 64.8				
Day 7	43.5 ± 10.8	3.31 ± 1.58	627 ± 164	2.22 ± 0.27	47.3 ± 24.9	339 ± 90.4	23,200 ± 13,200

Study 004							
200 mg/day (*n* = 15)—day 7	51.6 ± 14.5	3.41 ± 1.18	695 ± 200		38.6 ± 5.3		
500 mg/day (*n* = 11)—day 7	209 ± 72.7	4.46 ± 1.57	3140 ± 1195		37.5 ± 2.8		
750 mg/day (*n* = 6)—day 7	328 ± 68.6	3.33 ± 0.52	4810 ± 1522		38.6 ± 4.1		
1000 mg/day (*n* = 3)—day 7	543 ± 60.5	4.33 ± 1.53	8327 ± 911		41.6 ± 5.9		

All values reported as arithmetic means ± standard deviation (SD)

*SAD* single ascending dose, *MAD* multiple ascending dose, *NR* no result, *C*_*max*_ maximum concetration, *AUC* area under the concentration-time curve,* t*_*1/2*_ half-life, *V*_*z*_*/F* volume of distribution, *R*_*ac*_ accumulation ratio, *CL/F* clearance, *t*_*max*_ time to reach maximum cocentration, *ss* steady state

^a^Study drugs were administered in the fasted condition unless otherwise indicated

^b^One subject was excluded from descriptive statistics due to emesis after dosing

^c^One subject was excluded from descriptive statistics on Day 1 due to emesis after dosing

^d^CL

^e^*V*_*Z*_

The original article has been corrected.

